# PFAPA: a single phenotype with genetic heterogeneity

**DOI:** 10.1186/1546-0096-10-S1-A86

**Published:** 2012-07-13

**Authors:** Sivia K Lapidus, Puja Chitkara, Peter W Kim, Ivona Aksentijevich, Elaine F Remmers, Henry Feder, Beverly K Barham, Anne Jones, Michael M Ward, Karyl S Barron, Daniel L Kastner, Silvia Stojanov

**Affiliations:** 1 Children's University Hospital Munich, Munich, Bavaria, Germany; 2 Goryeb Children's Hospital, Maplewood, NJ, USA; 3 NHGRI, Damascus, MD, USA; 4 NHGRI, Bethesda, MD, USA; 5 NHGRI NIH, Bethesda, MD, USA; 6 NIAID, Bethesda, MD, USA; 7 NIH, Bethesda, MD, USA; 8 NIH/NHGRI, Bethesda, MD, USA; 9 Connecticut Health Sciences Center/Connecticut Children's Medical Center, Farmington, CT, USA; 10 SDAMC, San Diego, CA, USA

## Purpose

PFAPA (periodic fever, aphthous stomatitis, pharyngitis and cervical adenitis) is the most common recurrent fever syndrome affecting children. No infectious or genetic etiology has been identified. We aimed to compare various clinical characteristics between sporadic and familial cases in addition to describing the heritability of familial cases.

## Methods

PFAPA patients were prospectively recruited. All patients had genetic testing to exclude mutations in the known fever genes (*MVK*, *MEFV*, *TNFRSF1A*, *NLRP3*, and *ELA2*). These PFAPA patients have been classified as sporadic or familial cases based on family history. Familial cases included those with a family member having PFAPA or a family member having a feature of PFAPA (recurrent fever, oral ulcer, pharyngitis, or lymphadenopathy). The demographics, symptoms, response to therapies, and clinical characteristics were compared for sporadic and familial cases. Detailed histories were obtained from families with multiple members affected by PFAPA.

## Results

Eighteen of 45 PFAPA patients (40%) had no family history of PFAPA features, nine of 45 patients (20%) had at least one family member with PFAPA, and 18 of 45 patients (40%) had a family history of PFAPA symptoms. Sporadic and familial PFAPA patients did not exhibit significant differences in demographics, height of fever, and defining as well as non-defining features of PFAPA. Five families had a total of 12 children affected with PFAPA, in addition to affected adult members. One of the five families had a pattern consistent with autosomal recessive inheritance, and four had patterns resembling autosomal dominant inheritance.

## Conclusion

The sporadic and familial cases of PFAPA in whom monogenic recurrent fever syndromes have been excluded do not appear to differ in their features or severity of disease. The familial cases of PFAPA imply a possible genetic propensity to this condition with an uncertain mode of inheritance, the elucidation of which may direct genetic studies for this common autoinflammatory condition.

## Disclosure

Sivia K. Lapidus: None; Puja Chitkara: None; Peter W. Kim: None; Ivona Aksentijevich: None; Elaine F. Remmers: None; Henry Feder: None; Beverly K. Barham: None; Anne Jones: None; Michael M. Ward: None; Karyl S. Barron: None; Daniel L. Kastner: None; Silvia Stojanov: None.

